# Don't forget the oldies: using IoT to connect the legacy medical equipments

**DOI:** 10.1186/s13054-022-04112-y

**Published:** 2022-08-25

**Authors:** Linghua Yu, Ming Yao

**Affiliations:** Gastroenterology and Hepatology Department, Institute of Liver Diseases, The Affiliated Hospital of Jiaxing University, 1882 Central-South Road, Jiaxing, 314001 Zhejiang People’s Republic of China

**Keywords:** Medical equipment, Medical negligence, Intensive care unit

Medical devices have been evolving for decades, but there's still a long way to go. According to studies, an estimate of nearly 100,000 annual deaths by medical negligence in the USA, making them the third leading cause of patient death in the country, behind only heart disease and cancer [1, 2]. Those events most likely occurred in the intensive care unit (ICU). The major causes of medical negligence include medication errors (20%), diagnostic errors (17%), and failure to prevent infection (12%), all of which could be avoided by improving the synergy between medical devices [3]. Thus, an essential step in reducing medical errors is to connect medical devices and share data seamlessly with each other.

The most effective way to coordinate medical equipment is through using a universal standard communication interface. The Internet of Things (IoT) promises seamless connectivity between all devices, allowing for more accurate and up-to-date health information exchange for patients. By connecting these often disparate pieces of equipment, we can reduce medical errors and save lives. For example, imaging an insulin pump connected to a glucose monitor, the pump will be able to adjust insulin levels without human intervention.

Unfortunately, the medical industry is at a very awkward stage in the application of IoT technology, and medical staff act as a "human network" to do all the connection and coordination work [4]. In an ICU, there are ventilators, monitors, infusion pumps, defibrillators and other devices that require communication between them. The problem is that each one has its own proprietary interface and cannot communicate with others unless they are made by the same manufacturer. At present, most medical devices are connected to computers by point-to-point topology through serial interfaces [5]. If the computer needs to be connected to other medical equipment, rewiring is required. We can see a huge gap exists between the cutting-edge technology and old medical equipment in the hospital.

Wireless communication makes the interconnection between medical equipment simple, flexible, and reliable. However, a majority of existing medical devices only support serial interfaces and can't run the wireless network. Herein, we designed a device to achieve wireless interconnection for old medical equipment based on IoT technology. This device is plugged into the serial interface of medical equipment and transfers the data to the wireless network. New technology plus a small device, could rescue these oldie devices by allowing them to share data seamlessly over a wirelessly connected network (Fig. 1). We hope this will help prevent future medical errors and save lives!

**Fig. 1:**
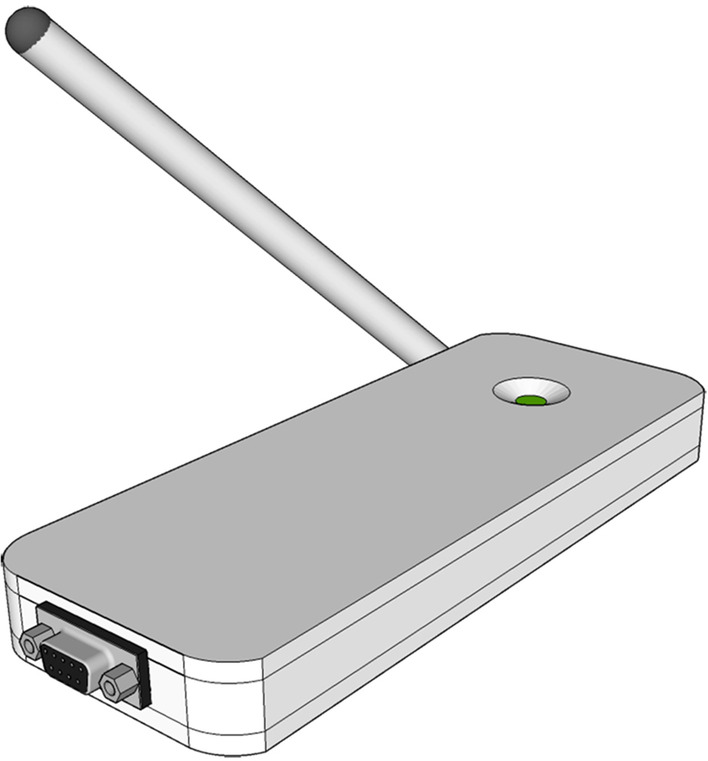
IoT device we designed

## Data Availability

Not applicable.
